# Evaluation of the Accuracy of Intraocular Lens Power Calculation Formulas for Cataract Surgery in Patients with Keratoconus

**DOI:** 10.18502/jovr.v20.17679

**Published:** 2025-11-10

**Authors:** Firouze Hatami, Sina Khosravi Mirzaei, Mohammad Ali Javadi, Sepehr Feizi, Sare Safi, Seyed Bagher Hosseini

**Affiliations:** ^1^Ophthalmic Research Center, Research Institute for Ophthalmology and Vision Science, Shahid Beheshti University of Medical Sciences, Tehran, Iran; ^2^Ocular Tissue Engineering Research Center, Research Institute for Ophthalmology and Vision Science, Shahid Beheshti University of Medical Sciences, Tehran, Iran; ^3^Ophthalmic Epidemiology Research Center, Research Institute for Ophthalmology and Vision Science, Shahid Beheshti University of Medical Sciences, Tehran, Iran

**Keywords:** Cataract Surgery, Intraocular Lens Power Calculation Formula, Keratoconus

## Abstract

**Purpose:**

To compare the refractive accuracy of different intraocular lens (IOL) power calculation formulas in eyes with keratoconus (KCN) undergoing cataract surgery.

**Methods:**

This retrospective case series included the medical records of patients with KCN who underwent optical biometry and cataract surgery with IOL implantation. The predicted spherical equivalent (SE) values were calculated using the Holladay 1, Hoffer Q, SRK/T, and SRK II formulas. Additionally, a subgroup analysis was performed for eyes with available data on anterior chamber depth to compare the accuracy of Haigis, Barrett Universal II, Barrett True-K, EVO 2.0, Kane, and Kane KCN formulas. The mean prediction error (PE), mean absolute error (MAE), median absolute error, and the percentage of eyes within a PE of ± 0.25 diopters (D), ± 0.50 D, ± 0.75 D, and ± 1.00 D were calculated.

**Results:**

Forty-seven eyes of 30 patients were included. The MAE was significantly different among the Holladay 1, Hoffer Q, SRK/T, and SRK II formulas. The Holladay 1 and Hoffer Q formulas led to a hyperopic refractive shift. The SRK/T and SRK II formulas tended toward a myopic refractive outcome. The MAE was lowest for the SRK/T formula (0.39 D), followed by the Holladay 1 (0.48 D), Hoffer Q (0.59 D), and SRK II (0.87 D). Statistical analysis revealed a significantly lower MAE for the SRK/T formula compared to the Hoffer Q and SRK II formulas (*P* < 0.05). The percentage of eyes within a PE of ± 0.50 D was 70.2% for SRK/T, 57.44% for Holladay 1, 48.93% for Hoffer Q, and 29.78% for SRK II. The subgroup analysis comprising 11 eyes showed no significant difference among six other formulas (Haigis, Barrett Universal II, Barrett True K, EVO 2.0, Kane, and Kane KCN), with Barrett True-K having the least MAE.

**Conclusion:**

The SRK/T was the most accurate IOL power calculation formula in this study, and Holladay 1 could be an alternative choice. SRK II had the lowest accuracy in predicting refractive outcomes. Among modern formulas, Barrett True-K demonstrated the highest accuracy in eyes with KCN.

##  INTRODUCTION

Keratoconus (KCN) is a progressive non-inflammatory corneal ectasia leading to central or paracentral corneal thinning and reduction in visual acuity.^[1,2]^ Individuals with KCN often experience an earlier onset of cataracts compared to those with normal eyes.^[3]^ The surgical management of cataracts in patients with KCN is more challenging due to the corneal changes, making the accurate prediction of refractive outcomes notably more difficult than in normal eyes.^[4,5]^

The refractive outcomes following intraocular lens (IOL) implantation in non-KCN eyes typically demonstrate a high level of satisfaction, with 72% to 80% of eyes achieving refractive error within 0.5 diopters (D),^[6]^ while the results for eyes with KCN are often less favorable.^[7]^ The IOL power calculation formulas are designed for normal eyes, and using them for eyes with KCN could cause errors in postoperative refraction. This difference in outcomes could have several reasons. First, IOL power calculation formulas assume the ratio of the anterior to posterior corneal power based on established eye models, which are not designed for eyes with KCN.^[8]^ Second, effective lens position (ELP) estimation is calculated using the corneal power; therefore, false corneal power calculation leads to inaccurate ELP.^[9]^ Third, traditional keratometry measurements rely on the assumption of a constant corneal curvature along both the flat and steep meridians; however, this assumption may be inaccurate in keratoconic eyes, where irregularities in corneal curvature and variability in a particular meridian make the principal power meridian measurements non-orthogonal.^[10,11]^

Various approaches have been suggested to improve outcomes in eyes with KCN. These include targeting additional myopia^[12]^ and measuring the anterior and posterior corneal curvature using Scheimpflug tomography and then converting these values to an equivalent keratometry reading that can be used in selected formulas.^[13]^ Identifying the most accurate formula may further contribute to optimizing outcomes. Previous studies have identified different formulas, such as SRK/T,^[14,15]^ Kane KCN,^[11]^ Barrett Universal II,^[16]^ and Barrett True K,^[17]^ as being more accurate than others. Older formulas, such as SRK II, have also been reported to yield better results in mild KCN.^[18]^ Considering that different formulas employ different factors to estimate desired target refraction, it is important to assess the accuracy of their prediction.

In this study, we aimed to compare different IOL power calculation formulas to find the most accurate formula in predicting refractive outcomes in patients with KCN.

##  METHODS

This retrospective consecutive case series adheres to the tenets of the Declaration of Helsinki and was approved by the Research Committee of Ophthalmic Research Center affiliated with Shahid Beheshti University of Medical Sciences, Tehran, Iran (approval number: IR.SBMU.ORC.REC.1403.001). All cataract surgeries were performed by a single surgeon (MAJ) from March 2015 to July 2023 at a single center.

All patients were diagnosed with KCN and cataract through clinical examination and corneal topography. Patients with previous ocular surgery, history of ocular inflammation, glaucoma, diabetic retinopathy, macular disorders, and intraoperative or postoperative complications were excluded. Before surgery, a comprehensive examination was performed, including assessment of best-corrected visual acuity (BCVA), intraocular pressure, standard keratometry, subjective manifest refraction, and fundoscopy. Biometric values, including keratometry values and axial length measurements, were obtained using IOLMaster 500 optical biometer (software version 7.5; Carl Zeiss Meditec AG, Jena, Germany). In addition, the severity of KCN was defined in three stages. Eyes with a mean central keratometry of ≤ 48 D, between 48 and 53 D, and > 53 D were classified as stage 1, stage 2, and stage 3, respectively.^[15,19]^ Eyes with unmeasurable refraction, corneal radii > 55, corneal scar, corneal perforation, or corneal thickness < 200 µm were considered as stage 4 KCN and were excluded from this study.^[19]^

All patients underwent sutureless cataract surgery and IOL implantation within the capsular bag through a 2.8-mm corneal tunnel incision. Postoperative care included chloramphenicol 0.5% eye drops administered every 6 hours for 1 week, and betamethasone 0.1% eye drops initiated every 3 hours and gradually tapered over 4–6 weeks. All patients were followed up for a minimum of 1 month. At the postoperative follow-ups, patients were first examined by an ophthalmologist. Then, a certified optometrist measured subjective refraction (spherical equivalent [SE], astigmatism, and axis orientation) and BCVA of all patients using a Snellen chart.

### IOL Power Calculation

The Holladay 1,^[20]^ Hoffer Q,^[21]^ SRK/T,^[22]^ and SRK II^[23,24]^ formulas were obtained by inputting the required values into Excel software (Microsoft Corp, Redmond, WA, USA). Furthermore, for eyes with available anterior chamber depth (ACD) data, the accuracy of six newer formulas, including Haigis,^[25]^ Barrett Universal II,^[26]^ Barrett True-K,^[27]^ EVO 2.0,^[28]^ Kane,^[29]^ and Kane KCN,^[29]^ was evaluated using Excel or online calculators. While different types of IOLs were implemented, optimized lens constants provided by the User Group for Laser Interference Biometry (ULIB) were used.^[15,30]^

The prediction error (PE) for each formula was considered as the actual postoperative SE refraction minus the predicted SE refraction. A negative PE signifies a greater postoperative myopia compared to the anticipated refraction, while a positive PE indicates a more hyperopic outcome. The mean PE, its standard deviation (SD), the mean absolute error (MAE), and the median absolute error (MedAE) were calculated. In addition, the percentage of eyes with a PE within ± 0.25 D, ± 0.50 D, ± 0.75 D, and ± 1.00 D was evaluated.

### Statistical Analysis 

All statistical analyses were performed using SPSS software (IBM Corp. Version 27.0, Armonk, NY, USA). The normality of data distribution was assessed with the Shapiro-Wilk test. Due to the non-normal distribution of data, the Friedman test was used to determine the statistical significance between the MAE of each formula. Then, the Wilcoxon signed-rank test was performed to compare formulas with each other. To calculate the percentage of eyes within ± 0.50 D and ± 1.00 D ranges, the Cochran's Q test with Bonferroni correction was used. For all analyses, a *P*-value < 0.05 was considered statistically significant.

##  RESULTS

A total of 47 eyes from 30 patients (16 males) with a mean age of 60.89 ± 9.49 years were included in this study. Table 1 shows the demographics, baseline clinical characteristics, and ocular biometry data. Six IOL types were used for patients: Alcon AcrySof SN60WF (23 eyes), Alcon AcrySof SA60AT (12 eyes), Alcon AcrySof Toric SN6AT (2–9) (4 eyes), Alcon AcrySof MA60AC (1 eye), Bausch & Lomb enVista toric MX60T (4 eyes), and Bausch & Lomb enVista MX60P (3 eyes). The constants used for each IOL model are displayed in Table 2. The mean postoperative SE was –0.50 ± 0.40 D. After cataract surgery, the mean BCVA improved in all eyes and reached 0.21 ± 0.10 logMAR (logarithm of the minimum angle of resolution). Thirty-two eyes were classified as stage 1 KCN, and fifteen eyes as stage 2. None of the included eyes belonged to stage 3.

Table 3 shows the refractive PE for each formula. The SRK/T and SRK II formulas resulted in a negative mean PE, indicating a tendency toward a myopic error. On the other hand, Holladay 1 and Hoffer Q had a positive mean PE, showing a hyperopic tendency for prediction. The Friedman test showed that there was a significant difference among all four formulas (*P* < 0.001). SRK/T had the lowest MAE, and the difference was statistically significant compared to the values obtained by Hoffer Q and SRK II (*P* < 0.05), but there was no significant difference between the SRK/T and the Holladay 1 (*P *= 0.16). Similarly, Holladay 1 had a significantly lower MAE compared to the Hoffer Q and the SRK II formulas. A significant correlation was also found between Hoffer Q and SRK II (*P* = 0.014). SRK II predicted the postoperative SE with the highest MAE, which was significantly less accurate than those obtained by all other formulas.

**Figure 1:**
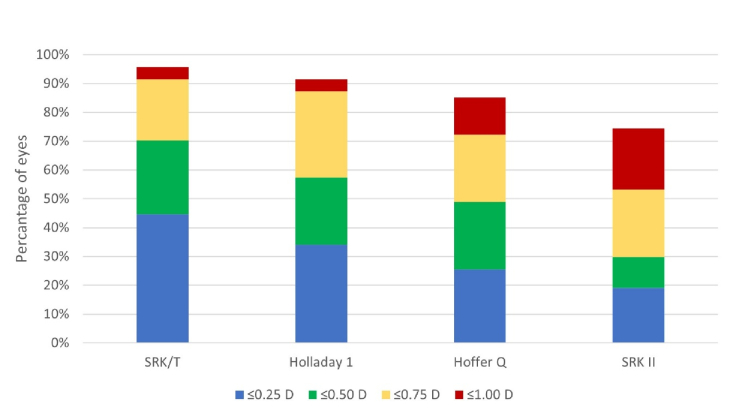
Stacked histograms comparing the percentage of eyes within different diopter (D) ranges of absolute prediction error for spherical equivalent (traditional formulas).

Figure 1 shows the percentage of eyes within PE of ± 0.25 D, ± 0.50 D, ± 0.75 D, and ± 1.00 D for Holladay 1, Hoffer Q, SRK/T, and SRK II formulas. The PE within ± 0.50 D was calculated in 33 eyes (70.2%) with SRK/T, 27 eyes (57.44%) with Holladay 1, 23 eyes (48.93%) with Hoffer Q, and 14 eyes (29.78%) with SRK II. Comparing the percentage of eyes within ± 0.50 D, we found significant differences between the SRK/T and SRK II formulas (*P* < 0.001) and the Holladay 1 and SRK II formulas (*P* = 0.023). Moreover, within the ± 1.00 D range, the SRK/T and Holladay 1 formulas had a significant difference with the SRK II (*P *= 0.003 and P = 0.031, respectively). Other comparisons between formulas showed insignificant differences.

**Figure 2:**
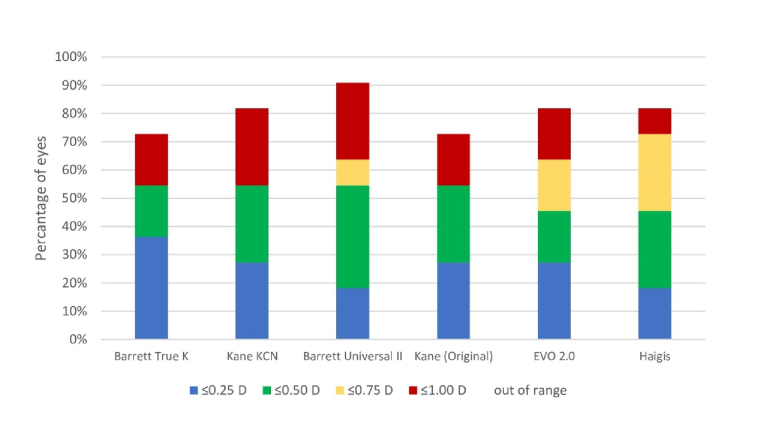
Stacked histograms comparing the percentage of eyes within different diopter (D) ranges of absolute prediction error for spherical equivalent (modern formulas).

Due to the unavailability of ACD for some eyes, the postoperative SE prediction of Haigis, Barrett Universal II, Barrett True-K, EVO 2.0, Kane, and Kane KCN formulas were performed on a sample size of 11 eyes (seven patients). Among these eyes, four types of IOL were implemented, including Alcon AcrySof SN60WF (two eyes), Alcon AcrySof Toric SN6AT (2–9) (two eyes), Bausch & Lomb enVista toric MX60T (four eyes), and Bausch & Lomb enVista MX60P (three eyes). Seven eyes were classified as stage 1, and four eyes as stage 2 KCN. Table 4 lists the refractive PE of the Haigis, Barrett Universal II, Barrett True-K, EVO 2.0, Kane, and Kane KCN formulas. The Barrett True-K and Kane KCN exhibited a negative mean PE, whereas the other four formulas demonstrated a positive mean PE. The Friedman test revealed no statistically significant difference in the MAE between these formulas (*P* = 0.735). Barrett True-K demonstrated the lowest MAE (0.54 D), while Haigis exhibited the highest MAE (0.68 D). The proportion of eyes within a PE of ± 0.50 D was 54.5% for the Barrett True-K, Kane KCN, Barrett Universal II, and Kane formulas. For a PE of ± 1.00 D, the Barrett Universal II demonstrated the highest proportion of eyes, at 90.9% [Figure 2].

**Table 1:** Demographics, baseline clinical characteristics, and ocular biometry data of patients

	**Mean**	**SD**	**Range**
Age (year)	60.89	9.49	44 to 77
Axial length (mm)	23.89	1.61	21.35 to 26.96
Keratometry (flat) (D)	45.22	2.32	40.61 to 49.20
Keratometry (steep) (D)	48.28	2.41	43.28 to 52.33
Mean keratometry (D)	46.75	2.22	42.26 to 50.44
IOL power (D)	17.23	4.28	4.00 to 23.00
Preoperative spherical equivalent (D)	–2.61	3.44	–11.25 to 2.25
Preoperative BCVA (logMAR)	0.51	0.35	0.15 to 2.00
D, diopters; IOL, intraocular lens; SD, standard deviation; BCVA, best corrected visual acuity; logMAR, logarithm of the minimum angle of resolution			

**Table 2:** Constants used for each intraocular lens model

**IOL model**	**A-Constant **	**pACD**	**sf**	**a0**	**a1**	**a2**
Alcon AcrySof SN60WF	119.0	5.64	1.84	–0.769	0.234	0.217
Alcon AcrySof SA60AT	118.8	5.44	1.67	–0.111	0.249	0.179
Alcon AcrySof Toric SN6AT	119.2	5.81	1.98	–0.323	0.213	0.208
Alcon AcrySof MA60AC	119.2	5.67	1.90	0.229	0.011	0.205
Bausch & Lomb enVista toric MX60T	119.2	5.68	1.91	1.46	0.400	0.100
Bausch & Lomb enVista MX60P	119.2	5.68	1.91	1.46	0.400	0.100

**Table 3:** Refractive prediction errors of traditional formulas for all included eyes (sorted by mean absolute error)

**Formula**	**MAE**	**MPE**	**PE SD**	**MedAE**
SRK/T	0.39	–0.14	0.47	0.29
Holladay 1	0.48	0.24	0.59	0.43
Hoffer Q	0.59	0.41	0.63	0.54
SRK II	0.87	–0.77	0.82	0.72
MAE, mean absolute error; MPE, mean prediction error; MedAE, median absolute prediction error; PE SD, standard deviation of the prediction error (all values are expressed in diopters).				

**Table 4:** Refractive prediction errors of modern formulas for 11 eyes (sorted by mean absolute error)

**Formula**	**MAE**	**MPE**	**PE SD**	**MedAE**
Barrett True-K	0.54	–0.27	0.65	0.31
Kane KCN	0.56	–0.04	0.70	0.45
Barrett Universal II	0.63	0.12	0.82	0.37
Kane (Original)	0.65	0.04	0.87	0.45
EVO 2.0	0.67	0.15	0.88	0.52
Haigis	0.68	0.20	0.87	0.61
MAE, mean absolute error; MPE, mean prediction error; MedAE, median absolute prediction error; PE SD, standard deviation of the prediction error; KCN, keratoconus (all values are expressed in diopters).				

##  DISCUSSION

Our study investigated the accuracy of IOL power calculation formulas in patients diagnosed with KCN. The SRK/T formula had the lowest MAE, indicating its superior accuracy compared to other formulas. The SRK II formula, with the highest MAE, was the least accurate. Among the six newer formulas (Haigis, Barrett Universal II, Barrett True K, EVO 2.0, Kane, and Kane KCN), Barrett True-K yielded the lowest MAE. The SRK/T formula demonstrated superior accuracy, with 70% of eyes achieving a PE within ± 0.50 D, while in normal eyes, this rate is approximately 75%.^[6,31]^ Notably, previous studies reported that the highest percentage of eyes achieving a PE within ± 0.50 D was 44% and 50% for patients with KCN.^[11,15]^ In KCN, the predictive accuracy of all formulas further decreases as ectasia severity progresses.^[16]^ The reduced accuracy in KCN can be attributed to several factors. First, IOL calculation formulas are primarily designed for patients with normal anterior and posterior corneal curvature, and IOL calculation formulas heavily rely on anterior corneal power while estimating posterior corneal power. Implementing these formulas for eyes with KCN that have asymmetry in corneal curvatures from early stages could reduce their final accuracy.^[32]^ Additionally, in higher stages of KCN, the ratio between the anterior and posterior curvatures could be even more disrupted, highlighting the importance of posterior curvature measurement.^[32]^ Heath et al^[17]^ showed that integrating total keratometry, rather than simply standard keratometry, improves the accuracy of IOL calculation formulas in patients with KCN. For all formulas in which both standard keratometry and total keratometry were available, applying total keratometry resulted in a lower MAE for each formula. Second, using the standard keratometric index (1.3375) for these patients may also lead to inaccurate IOL power estimations.^[33,34]^

In line with our findings, Savini et al^[15]^ and Kamiya et al^[35]^ found that SRK/T had the lowest MAE and the highest percentage of eyes within ± 0.50 D of PE (44% and 36 %, respectively). In another study, the superiority of the SRK/T over the Holladay 1, Hoffer Q, and SRK II formulas has been shown.^[14]^ However, these studies^[14,15][35]^ reported a positive MPE for SRK/T. This might be related to the inclusion of severe KCN cases in their studies, whereas our investigation focused on stages 1 and 2 of KCN. Studies suggest a correlation between increased KCN severity and higher PE, potentially leading to a hyperopic shift.^[35,36]^ Kane et al^[11]^ found a negative MPE for SRK/T in mild cases of KCN. Additionally, among IOL power calculation formulas (Barrett Universal II, Haigis, Hoffer Q, Holladay 1, Holladay 2, Kane, and SRK/T), the SRK/T formula showed the lowest tendency toward hyperopic refractive surprise. As described by Hagis et al^[37]^ and Sheard et al,^[38]^ SRK/T has a tendency toward myopic PE, and this could balance the hyperopic tendency seen in patients with KCN. Notably, studies have also shown that SRK/T is the most accurate formula in long eyes.^[21]^ Although in our study, only eight patients (17.02%) had an AL of > 26 mm, the SRK/T was the most accurate formula.

In our study, Holladay 1 resulted in a higher MAE compared to SRK/T, but the difference between these two formulas was not significant, suggesting that Holladay 1 may also be a reliable option for IOL power calculation in patients with KCN. Kamiya et al^[35]^ reported that among several IOL calculation formulas, Holladay 1 exhibited the second lowest MAE compared to SRK/T, with no statistically significant difference noted. Additionally, they identified a significant difference between the SRK/T and Hoffer Q formulas, which is consistent with our research findings. Similar studies have also found higher MEA for Holladay 1 and Hoffer Q compared to SRK/T.^[11,15][16][17]^

Kamiya et al^[35]^ identified the SRK II as having the highest MAE and a negative MPE. In another study conducted by Hashemi et al,^[14]^ SRK II had a higher MEA compared to SRK/T in all stages of KCN. However, Thebpatiphat et al^[5]^ reported the SRK II formula to be more accurate for IOL power calculation in mild KCN compared to the SRK/T formula. Nevertheless, their study included only five eyes, limiting the generalizability of their findings due to insufficient sample size for statistical analysis.

A few studies have compared the accuracy of Haigis, Barrett Universal II, EVO 2.0, and Kane formulas in patients with KCN. Wang et al^[16]^ showed the statistically significant superiority of the Barrett Universal II over Haigis in KCN stages 1 and 2. Similarly, Savini et al^[15]^ reported a lower MedAE with Barrett Universal II compared to Haigis. Another study found that the Barrett True-K had a lower MAE compared to the Kane, Kane KCN, Barrett Universal II, EVO 2, and Haigis formulas in eyes with severe and non-severe KCN.^[17]^

In another study by Kane et al,^[11]^ Kane KCN was the most accurate formula. In KCN stage 1, the Kane KCN formula had a significantly lower MAE than both Barrett Universal II and Haigis.^[11]^ In our study, Kane KCN also had a lower MAE compared to other modern formulas except Barrett True-K; however, no statistical significance was found. These differences in outcomes may be attributed to differences in KCN stages or biometric values, as well as differences in sample size. Additionally, another study reported that Barrett Universal II exhibited a lower MAE compared to the Kane and Haigis formulas among all patients,^[11]^ which aligns with our findings.

Our study had several limitations. First, the sample size was relatively small, and patients with stage 3 KCN were not included. Second, the use of different types of IOLs prevented the optimization of IOL constants due to the retrospective design of the study. Instead, we used the IOL constant from the ULIB website, which is recommended by Savini et al^[15]^ and Hoffer et al.^[39]^ Third, the unavailability of lens thickness measurements precluded the assessment of some formulas that require this parameter. Fourth, due to the limited availability of ACD, modern formulas were investigated with fewer cases. Moreover, the distribution of KCN stages was different in this subgroup compared with the total sample size. These factors hindered the comparison of traditional and modern formulas with each other. Fifth, our calculations relied solely on anterior corneal curvature, and incorporating total corneal power by measuring both anterior and posterior corneal curvatures might have enhanced the precision of our calculations.

In summary, the SRK/T formula was the most accurate IOL power calculation formula for patients with KCN. The Holladay 1 formula, with the second lowest MAE, was an alternative choice for these patients. Regarding newer formulas, Barrett True-K had the lowest MAE. However, our findings revealed lower predictive accuracy in KCN compared to normal eyes, indicating the challenge in IOL power calculation for KCN and the necessity for further investigation.

##  Financial Support and Sponsorship

None.

##  Conflicts of Interest

None.
